# The NSvc4 Protein of Rice Stripe Virus Suppresses Chloroplast-Mediated Defense by Interacting with NbPsbQ

**DOI:** 10.3390/ijms27093859

**Published:** 2026-04-27

**Authors:** Zongdi Li, Chenyang Li, Jianxiang Wu, Xiuling Yang, Xueping Zhou

**Affiliations:** 1 State Key Laboratory of Rice Biology and Breeding, Institute of Biotechnology, Zhejiang University, Hangzhou 310058, China; 11816056@zju.edu.cn (Z.L.); applne@hotmail.com (C.L.); wujx@zju.edu.cn (J.W.); 2 State Key Laboratory for Biology of Plant Diseases and Insect Pests, Institute of Plant Protection, Chinese Academy of Agricultural Sciences, Beijing 100193, China

**Keywords:** rice stripe virus, NSvc4, PsbQ, chloroplast, defense

## Abstract

The chloroplast, a key organelle for plant immunity, is frequently targeted by viral proteins to suppress host defense. Here, we demonstrate that NSvc4, the movement protein of rice stripe virus (*Tenuivirus oryzaclavatae*; genus *Tenuivirus*), functions as a chloroplast-localized virulence effector. We show that NSvc4 enters chloroplasts and directly associates with NbPsbQ, a subunit of the oxygen-evolving complex (OEC) of Photosystem II. This interaction competitively disrupts the binding of NbPsbQ to its native partners NbPsbO and NbPsbP, thereby dampening the accumulation of chloroplast-derived reactive oxygen species (cROS) and attenuating pathogen-triggered immune signaling. Genetic knockout of *NbPsbQ* enhanced plant susceptibility to RSV, confirming its role as a positive regulator of antiviral defense. Our study uncovers a distinct strategy whereby a viral movement protein inhibits chloroplast-mediated immunity by targeting extrinsic subunits of the OEC. These findings expand the functional scope of viral movement proteins and highlight the OEC as a critical battleground in plant–virus interactions.

## 1. Introduction

Long recognized for their role in photosynthesis, chloroplasts have emerged as central signaling hubs for plant immunity [1,2,3]. They act as major sites for the generation of reactive oxygen species (ROS), which function both as direct antimicrobial compounds and as key signaling molecules that activate defense-related gene expression [4,5,6]. Furthermore, chloroplasts are biosynthetic centers for key defense phytohormones, including salicylic acid (SA) and jasmonic acid (JA), which orchestrate local and systemic immune responses [7,8,9,10]. Chloroplasts-associated calcium signaling also contributes to pathogen perception and the activation of defense responses [5]. Therefore, maintaining the functional integrity of chloroplasts is vital for plant defense against diverse pathogens.

In the ongoing evolutionary arms race, plant viruses have evolved sophisticated strategies to subvert chloroplast-mediated defenses. A common tactic is the direct targeting of chloroplasts by viral effector proteins to suppress immune outputs. For instance, the C4 protein encoded by tomato yellow leaf curl virus (TYLCV) localizes to chloroplasts and interferes with the calcium-sensing receptor, thereby dampening SA-dependent defenses [11]. Likewise, the P1 protein encoded by turnip mosaic virus (TuMV) binds to and promotes the degradation of the chloroplast signal recognition particle cpSRP54, disrupting JA biosynthesis to promote viral infection [12]. Similarly, the geminiviral effector βC1 binds to the chloroplast-targeted host protein OSB1. This interaction impairs the OSB1-ALD1 interaction, leading to ALD1 destabilization and suppression of pipecolic acid biosynthesis, thereby attenuating chloroplast-mediated antiviral immunity [13]. Moreover, pepper mild mottle virus coat protein (CP) associates with the chloroplast outer envelope protein OMP24, interfering with its self-dimerization and thereby suppressing OMP24-induced stromule formation, perinuclear chloroplast clustering, and ROS accumulation, which are essential for antiviral defense [14].

Within the chloroplast, the photosynthetic apparatus, especially Photosystem II (PSII) together with its oxygen-evolving complex (OEC), is increasingly recognized not only as essential for energy conversion but also as a pivotal regulator and target of immune signaling [15,16,17]. OEC locates on the lumen-facing side of PSII and catalyzes the process of water oxidation to release electrons, protons, and molecular oxygen [18,19]. In higher plants, the stability of OEC is maintained by three extrinsic peripheral proteins, PsbO, PsbP, and PsbQ [20,21,22]. PsbO is vital for maintaining the stability of the manganese-cluster catalytic site, whereas PsbP and PsbQ optimize calcium and chloride ion concentrations to enable efficient oxygen evolution [23,24,25,26,27,28]. OEC has emerged as a target exploited by various pathogens [29,30,31]. For instance, the bacterial effector HopN1 from *Pseudomonas syringae* targets and degrades PsbQ to suppress PSII efficiency and the chloroplast-derived ROS (cROS)-dependent hypersensitive response [32]. In plant–virus interactions, multiple viral proteins directly engage OEC components to facilitate infection. Alfalfa mosaic virus (AMV) CP interacts with the PsbP protein, inhibiting its antiviral activity and chloroplast localization [33]. Binding of PsbP to βC1, the RNA silencing suppressor encoded by radish leaf curl betasatellite, hindering the ability of βC1 to sequester viral DNA and weakening host defense [34]. Furthermore, the TGB3 protein encoded by alternanthera mosaic (AltMV) targets PsbO, an interaction correlated with virus-induced chloroplast vesiculation and systemic necrosis [35]. These collective findings highlight OEC as a crucial interface where antiviral defenses are executed and subsequently subverted by viral counter-defenses.

Rice stripe disease caused by rice stripe virus (RSV, *Tenuivirus oryzaclavatae*; genus *Tenuivirus*; family *Phenuiviridae*) represents a major threat to rice yield and food safety across East Asia [36,37,38]. It is primarily transmitted via the small brown planthopper (*Laodelphax striatellus*) in a persistent-propagative mode [39]. Infection of rice plants with RSV leads to characteristic chlorotic stripes, necrosis of newly emerged leaves, and plant stunting [36]. RSV can also be mechanically inoculated onto the model plant *Nicotiana benthamiana*, where it induces veinal chlorosis, leaf curling, and dwarfing symptoms [40]. RSV possesses a segmented, negative-sense RNA genome consisting of four RNA segments (RNA1-RNA4) [37]. These segments encode seven viral proteins, including RNA-dependent RNA polymerase (RdRp), the nonstructural proteins NS2, NS3, and NSvc4, coat protein (CP), and disease-specific protein (SP) [37,40,41]. Previously, NSvc4 was demonstrated to function as a movement protein that localizes to plasmodesmata to facilitate viral cell-to-cell movement [42]. NSvc4 also localizes to chloroplasts and suppresses chloroplast-mediated defense to promote viral infection [40]. Although NSvc4 was shown to interact with NbPsbQ within chloroplasts [40], the molecular mechanism by which chloroplast-localized NSvc4 subverts host immunity via its interaction with NbPsbQ remains elusive.

In this study, we demonstrate that NSvc4 interacts with NbPsbQ, thereby interfering with the association of NbPsbQ with NbPsbO and NbPsbP. Consequently, cROS accumulation was decreased and the expression of defense-related genes was downregulated. Our findings reveal a virulence strategy in which a viral movement protein directly sabotages chloroplast defense machinery to promote infection, offering new perspectives on the molecular interplay between plants and viruses and highlighting potential targets for developing virus-resistant crops.

## 2. Results

### 2.1. Knocking Out of NbPsbQ Promotes RSV Infection

**Figure 1:**
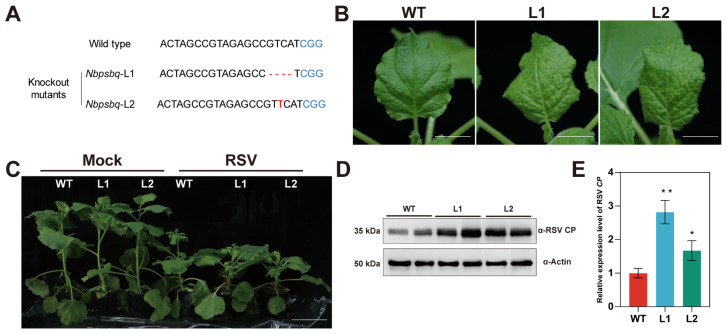
Knocking out of *NbPsbQ* in *N. benthamiana* promotes RSV infection. (**A**) Schematic representation of the frame shift mutations in *Nbpsbq*-L1 and *Nbpsbq*-L2 plants. Blue letters indicate the protospacer adjacent motif (PAM) sequence, while the red area marks the mutation type. (**B**) Symptoms of RSV-infected WT, *Nbpsbq*-L1 and *Nbpsbq*-L2 plants at 7 dpi. Scale bars = 2 cm. (**C**) Symptom of RSV-infected WT, *Nbpsbq*-L1 and *Nbpsbq*-L2 plants at 20 dpi. Scale bars = 10 cm. (**D**) Immunoblotting analysis of the RSV CP in WT, *Nbpsbq*-L1 and *Nbpsbq*-L2 plants inoculated with RSV at 7 dpi. (**E**) qRT-PCR analysis of the *CP* transcript in RSV-infected WT, *Nbpsbq*-L1 and *Nbpsbq*-L2 plants at 7 dpi. The relative abundance of viral RNA was calculated by normalization with the internal control *NbActin*. The accumulation of viral RNA in WT plants was set as 1. Statistical significance was assessed using Student’s *t*-test; asterisks indicate significant differences between WT and *Nbpsbq*-L1 or *Nbpsbq*-L2 (*n* = 3, * *p* < 0.05, ** *p* < 0.01).

Previously, we showed that RSV infection was enhanced in *NbPsbQ*-silenced *N. benthamiana* plants [40]. To better understand the function of NbPsbQ during RSV infection, we generated *NbPsbQ*-knockout *N. benthamiana* plants using CRISPR/Cas-mediated gene editing. Two independent knockout lines, *Nbpsbq*-L1 and *Nbpsbq*-L2, were obtained and showed growth phenotypes similar to wildtype (WT) *N. benthamiana* plants (Figure S1). Sequence analysis showed that *Nbpsbq*-L1 carried a four-nucleotide (nt) deletion at the cleavage cite, while *Nbpsbq*-L2 harbored a one-nt insertion (Figure 1A). We then challenged *Nbpsbq*-L1, *Nbpsbq*-L2, and WT plants with RSV by mechanical inoculation. At 7 days post-inoculation (dpi), *Nbpsbq*-L1 and *Nbpsbq*-L2 plants developed obvious vein yellowing symptom, while WT plants did not show any noticeable symptom (Figure 1B). By 20 dpi, although all the plants displayed typical symptom following RSV infection, *Nbpsbq*-L1 and *Nbpsbq*-L2 plants showed more severe dwarf symptom than WT plants (Figure 1C). Immunoblotting and quantitative real-time PCR (qRT-PCR) revealed that the levels of the *CP* transcript and CP were significantly higher in *Nbpsbq*-L1 and *Nbpsbq*-L2 plants than that in WT plants (Figure 1D,E), indicating that NbPsbQ negatively regulates RSV infection.

### 2.2. NbPsbQ Is Required for cROS Production During RSV Infection

**Figure 2:**
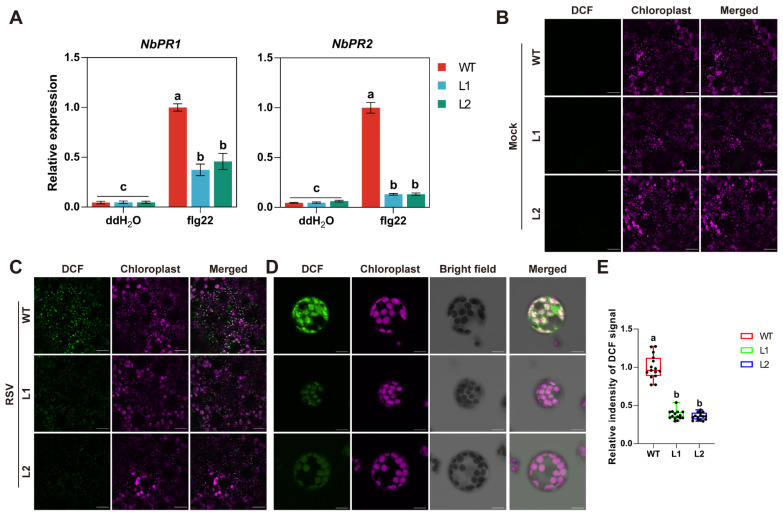
Knockout of *NbPsbQ* inhibits the expression of defense genes and the accumulation of chloroplast ROS (**A**) Relative expression levels of *NbPR1* and *NbPR2* measured by qRT-PCR in WT, *Nbpsbq*-L1 and L2 plants after infiltration with ddH_2_O or flg22. *NbActin* was used as an internal control, and the relative expression of defense genes in flg22-treated WT plants was set as 1. Data are presented as mean ± SD (*n* = 3). One-way ANOVA followed by Tukey’s HSD post hoc test was performed for multiple comparisons. Different letters indicate statistically significant differences between groups (*p* < 0.05). Each experiment was performed three times independently with comparable results. (**B**) Detection of ROS accumulation in *N. benthamiana* leaves. The upper leaves of WT, *Nbpsbq*-L1 and *Nbpsbq*-L2 plants were injected with 10 mM H_2_DCF-DA. One hour later, the distribution of DCF fluorescence was visualized using confocal microscopy. Scale Bars = 50 μm. (**C**) Detection of ROS accumulation in RSV-infected WT, *Nbpsbq*-L1 and *Nbpsbq*-L2 plants. Plants were injected with 10 mM H2DCF-DA. One hour later, the distribution of DCF fluorescence was visualized using confocal microscopy. Scale Bars = 50 μm. (**D**) Detection of ROS accumulation in protoplasts. A 10 mM quantity of H_2_DCF-DA was added to protoplasts isolated from RSV-infected *N. benthamiana* plants described in (**C**). One hour later, the DCF subcellular localization was observed under a confocal microscope. Scale Bars, 5 μm. (**E**) Quantification of DCF fluorescence intensity in chloroplasts shown in (**D**). The relative intensity of DCF signal was normalized to the value in WT. One-way ANOVA followed by Tukey’s HSD post hoc test was performed for multiple comparisons. Different letters indicate statistically significant differences between groups (*n* = 15, *p* < 0.05). Each experiment was performed three times independently with comparable results.

Given that NbPsbQ negatively regulates RSV infection, we investigated whether it is involved in basal immunity. We treated WT and *NbPsbq*-knockout plants with flg22, a bacterial flagellin peptide that triggers the host immune response, and then used qRT-PCR to detect the expression of defense-related genes *NbPR1* and *NbPR2*. Following flg22 treatment, the transcripts of *NbPR1* and *NbPR2* were much lower in *Nbpsbq*-L1 and *Nbpsbq*-L2 plants than in WT plants (Figure 2A), suggesting a potential role of NbPsbQ in basal immunity.

As NbPsbQ is a key component of the OEC, we wondered whether it is required for the cROS burst during RSV infection. To this end, we determined whether RSV infection boosts cROS production. We used the ROS-sensitive dye 2′7′-dichlorodihydrofluorescein diacetate (H_2_DCF-DA) to monitor the subcellular localization of ROS. Oxidized dichlorofluorescein (DCF) was monitored in various subcellular compartments of *N. benthamiana* leaves infected with RSV, including the nucleus, cytosol, and chloroplasts, while no DCF signal was detected in mock-inoculated leaves (Figure S2A). To better observe the accumulation of ROS in chloroplasts, protoplasts were purified from both mock-inoculated and RSV-infected leaves. Abundant DCF signal was detected in chloroplasts of RSV-infected leaves; however, no DCF signal was observed in protoplasts isolated from mock-inoculated WT leaves (Figure S2B). These results indicate that RSV infection induces ROS burst in *N. benthamiana* chloroplasts.

To determine whether NbPsbQ is required for cROS production, we examined cROS accumulation in *Nbpsbq*-L1 and *Nbpsbq*-L2 leaves under both mock-inoculated and RSV-infected conditions. In mock-inoculated *Nbpsbq*-L1, *Nbpsbq*-L2 leaves, the fluorescent signal from DCF was undetectable, similar to that in WT leaves (Figure 2B), suggesting that knockout of *NbPsbQ* does not affect basal ROS levels. Upon RSV infection, strong DCF signal was observed in WT leaves; however, the DCF fluorescent intensity was significantly reduced in *Nbpsbq*-L1 and *Nbpsbq*-L2 leaves (Figure 2C). A markedly reduced chloroplastic DCF signal was also observed in protoplasts isolated from RSV-infected *Nbpsbq*-L1 and *Nbpsbq*-L2 plants (Figure 2D,E). These data indicate that NbPsbQ is indispensable for cROS production in response to RSV infection.

### 2.3. NSvc4 Subverts Chloroplast-Mediated Immunity via Interaction with NbPsbQ

**Figure 3:**
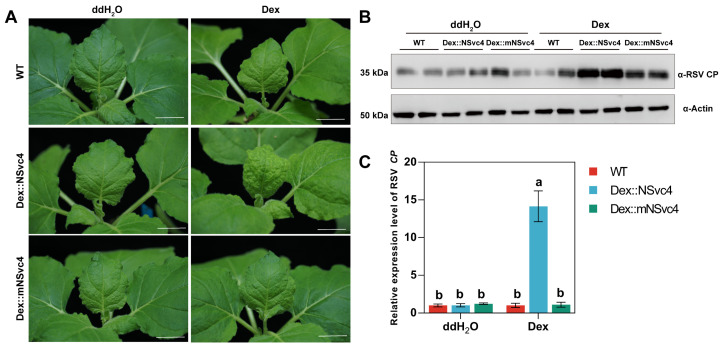
Dex-induced NSvc4 expression enhances RSV infection in *N. benthamiana* plants. (**A**) Symptoms of WT, Dex::NSvc4 and Dex::mNSvc4 plants treated with ddH_2_O or Dex at 7 days post-infection (dpi) of RSV. Scale bars = 2 cm. (**B**) Detection of RSV CP in WT, Dex::NSvc4 and Dex::mNSvc4 plants treated with ddH_2_O or Dex. Leaves of RSV-infected plants shown in (**A**) were subjected to total protein extraction and the accumulation of RSV CP was analyzed by Western blot. (**C**) Detection of viral RNA level in WT, Dex::NSvc4 and Dex::mNSvc4 plants treated with ddH_2_O or Dex. Leaves from the RSV-infected plants shown in (**A**) were subjected to total RNA extraction and the transcript level of RSV *CP* was determined by qRT-PCR. *NbActin* was used as an internal control for data normalization. Data are presented as mean ± SD (*n* = 3). One-way ANOVA followed by Tukey’s HSD post hoc test was performed for multiple comparisons. Different letters indicate statistically significant differences between groups (*p* < 0.05). Three independent experiments were performed, and the results from a representative experiment were shown.

We previously demonstrated that expression of the full-length NSvc4 can suppress the transcription of *PR* genes and cROS production upon immune activation [40]. To investigate the function of chloroplast-targeted NSvc4 in the suppression of chloroplast-mediated immunity, we generated transgenic *N. benthamiana* plants expressing NSvc4 or its non-chloroplast-localized mutant (mNSvc4) under a dexamethasone (Dex)-inducible promoter (Figure S3). After Dex induction and flg22 treatment, the expression of *NbPR1* and *NbPR2* and the generation of cROS were reduced in plants expressing full-length NSvc4 upon RSV infection (Figures S4 and S5). Moreover, Dex-induced expression of NSvc4 accelerated RSV infection in *N. benthamiana* plants (Figure 3A). Western blotting and qRT-PCR analyses revealed that virus accumulation was higher in these plants than in the control plants (Figure 3B,C). These results suggest that the chloroplast-targeted NSvc4 enhances the pathogenicity of RSV by inhibiting host immune response.

**Figure 4:**
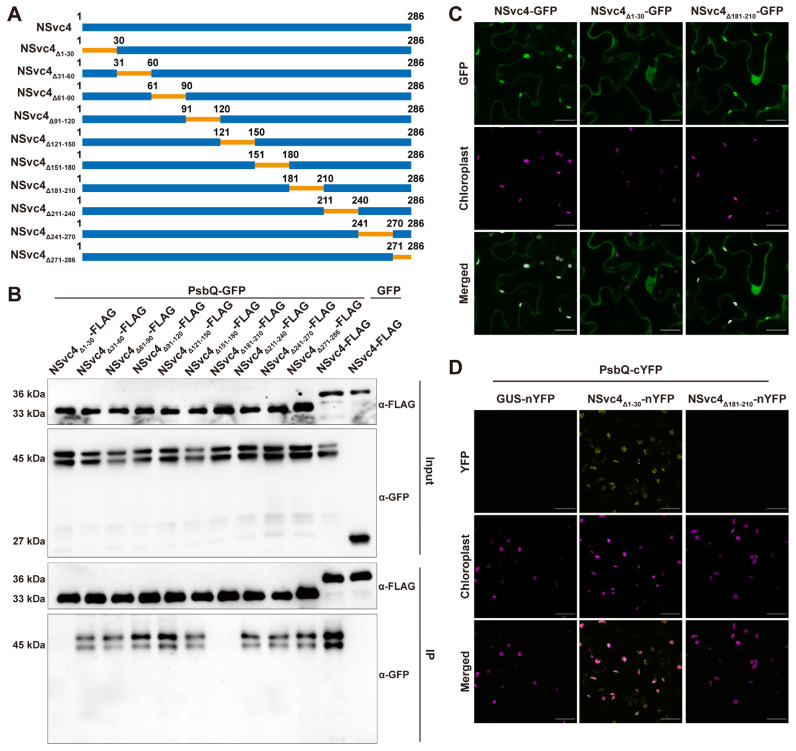
The 181–210 amino acids are essential for NSvc4 to interact with NbPsbQ. (**A**) Diagram of the construction of NSvc4 mutants. (**B**) Co-IP analysis of the interaction between NSvc4 mutants and NbPsbQ. (**C**) Confocal microscopy of the subcellular localization of NSvc4, NSvc4_Δ1–30_ and NSvc4_Δ181–210._ *N. benthamiana* leaves expressing NSvc4-GFP, NSvc4_Δ1–30_-GFP and NSvc4_Δ181–210_-GFP were observed at 48 hpi. Scale bars, 20 μm. (**D**) BiFC assays showing the interaction between NSvc4_Δ181–210_ and NbPsbQ. Scale bars, 20 μm.

To determine whether NSvc4-mediated suppression of host immunity is dependent on its interaction with NbPsbQ, we constructed ten deletion mutants of NSvc4, including NSvc4_Δ1–30_, NSvc4_Δ31–60_, NSvc4_Δ61–90_, NSvc4_Δ91–120_, NSvc4_Δ121–150_, NSvc4_Δ151–180_, NSvc4_Δ181–210_, NSvc4_Δ211–240_, NSvc4_Δ241–270_, NSvc4_Δ271–286_, to identify the key region of NSvc4 required for interaction with NbPsbQ (Figure 4A). Each NSvc4 mutant was fused at its C-terminus with a FLAG epitope tag, and coexpressed together with NbPsbQ-GFP in *N. benthamiana* leaves. Subsequently, coimmunoprecipitation (Co-IP) experiments were performed to assess the binding between these NSvc4 mutants and NbPsbQ. Co-IP assays showed that NbPsbQ-GFP was specifically immunoprecipitated with NSvc4-FLAG and eight NSvc4 mutants, while two mutants, NSvc4_Δ1–30_ and NSvc4_Δ181–210_, failed to interact with NbPsbQ (Figure 4B). To identify a NSvc4 mutant that loses the ability to interact with NbPsbQ while retaining chloroplast localization, we observed the subcellular localization of NSvc4_Δ1–30_ and NSvc4_Δ181–210_. Confocal microscopy revealed that NSvc4_Δ181–210_ exhibited a subcellular localization pattern similar to the full-length NSvc4, which accumulates in chloroplasts, whereas NSvc4_Δ1–30_ lacked chloroplast-localization (Figure 4C). Bimolecular fluorescence complementation (BiFC) assays showed that the YFP signals were detected exclusively in the combination of NSvc4-nYFP and NbPsbQ-cYFP, but not in the combination of NSvc4_Δ181–210_-nYFP and NbPsbQ-cYFP (Figure 4D), indicating that the 181–210 amino acids of NSvc4 are indispensable for its interaction with NbPsbQ.

**Figure 5:**
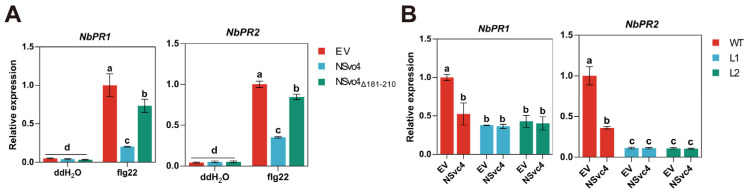
NSvc4 inhibits the expression of defense genes by interacting with NbPsbQ. (**A**) qRT-PCR analysis showing the relative transcript levels of *NbPR1* and *NbPR2* in *N. benthamiana* leaves infiltrated with EV, NSvc4, and NSvc4_Δ181–210_ and subsequent treated with flg22. *NbActin* was used for transcript normalizations. Different letters indicate significant differences (*p* < 0.001) based on one-way ANOVA (*n* = 3). (**B**) qRT-PCR analysis of the relative expression levels of *NbPR1* and *NbPR2* in WT and *NbPsbQ*-knockout (KO-*Nbpsbq*) plants expressing EV or NSvc4 after infiltration with flg22. *NbActin* was used as an internal control. Relative expression was calculated by setting the value of flg22-treated WT plants as 1. Different letters denote significant differences (*p* < 0.001) based on one-way ANOVA (*n* = 3). All experiments were repeated three times, yielding consistent results.

To test whether the 181–210 amino acids of NSvc4 are required for NSvc4 to impair host defense, *N. benthamiana* leaves transiently expressing empty vector (EV), NSvc4, and NSvc4_Δ181–210_ were treated with flg22 for 12 h, followed by qRT-PCR to measure the transcript levels of *NbPR1* and *NbPR2*. The transcripts of *NbPR1* and *NbPR2* were significantly lower in leaves expressing full-length NSvc4 than in leaves expressing EV or NSvc4_Δ181–210_ (Figure 5A). The leaves expressing NSvc4_Δ181–210_ showed slightly reduced expression levels of *NbPR1* and *NbPR2* compared with leaves infiltrated with EV, but this reduction was less pronounced than that observed in the leaves expressing the full-length NSvc4 (Figure 5A). We also transiently expressed EV and NSvc4 in *Nbpsbq* and WT plants, followed by flg22 treatment. qRT-PCR analysis showed that expression of NSvc4 could suppress the transcripts of *PR* genes in WT plants, but this suppression was abolished in *Nbpsbq* plants (Figure 5B). Taken together, these results demonstrate that NSvc4-mediated suppression of host immunity is dependent on its interaction with NbPsbQ.

### 2.4. NSvc4 Interferes with the Interaction Between NbPsbQ and NbPsbO, NbPsbP

OEC is generally surrounded by PsbO, PsbP, and PsbQ. In spinach, PsbQ has been shown to interact with PsbO and PsbP [24,43,44,45]. Since the extrinsic proteins of the OEC are highly conserved in higher plants [46], we tested whether NbPsbQ interacts with NbPsbO and NbPsbP in *N. benthamiana*. Co-IP assays revealed that both NbPsbO-Myc and NbPsbP-Myc could be specifically immunoprecipitated by NbPsbQ-FLAG (Figure S6A,B). BiFC experiments revealed that simultaneous expression of NbPsbQ-cYFP with NbPsbO-nYFP or NbPsbP-nYFP in *N. benthamiana* leaves reconstituted YFP fluorescence appearing as punctate spots in chloroplasts (Figure S6C). These results suggest that NbPsbQ interacts with NbPsbO and NbPsbP in vivo.

**Figure 6:**
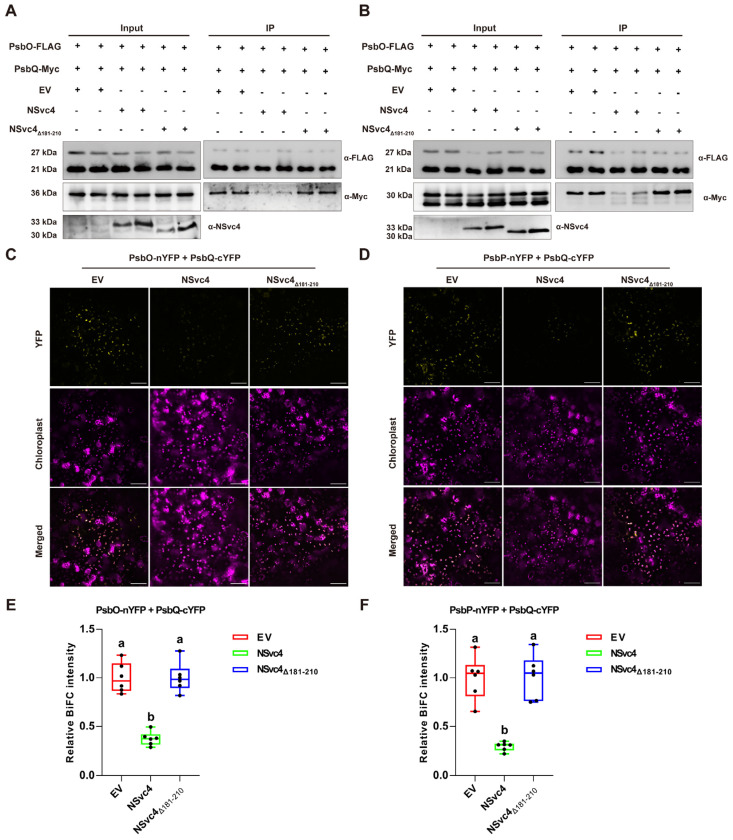
NSvc4 perturbs the interaction of NbPsbQ with NbPsbO and NbPsbP. (**A**) Co-IP analysis examining the interaction among NSvc4, NbPsbO, and NbPsbQ. (**B**) Co-IP analysis of the association among NSvc4, NbPsbP, and NbPsbQ. (**C**) BiFC was analysis of the influence of NSvc4 on the interaction between NbPsbO and NbPsbQ. Scale bars = 50 μm. (**D**) BiFC analysis of the influence of NSvc4 on the interaction between NbPsbP and NbPsbQ. Bars = 50 μm. (**E**) Quantification of YFP fluorescence intensity in (**C**). The relative intensity of the YFP signal was normalized to that of EV. One-way ANOVA followed by Tukey’s HSD post hoc test was performed for multiple comparisons. Different letters indicate statistically significant differences between groups (*n* = 6, *p* < 0.05). Three independent replicates were performed with comparable results. (**F**) Quantification of YFP fluorescence intensity in (**D**). The relative intensity of the YFP signal was normalized to that of EV. One-way ANOVA followed by Tukey’s HSD post hoc test was performed for multiple comparisons. Different letters indicate statistically significant differences between groups (*n* = 6, *p* < 0.05). Three independent replicates were performed and comparable results were obtained.

To understand the potential mechanism by which NSvc4 inhibits chloroplast-mediated immunity through their interaction, we determined whether NSvc4 could compete with NbPsbO and NbPsbP for NbPsbQ. We co-expressed NbPsbQ-FLAG + NbPsbO-Myc + EV, NbPsbQ-FLAG + NbPsbO-Myc + NSvc4, and NbPsbQ-FLAG + NbPsbO-Myc + NSvc4_Δ181–210_ in *N. benthamiana* leaves, respectively. Co-IP analysis demonstrated that the presence of NSvc4 substantially reduced the abundance of NbPsbO-Myc co-immunoprecipitated with NbPsbQ-FLAG (Figure 6A). However, co-expression of NSvc4_Δ181–210_ did not affect the NbPsbQ-NbPsbO interaction, as the amount of co-immunoprecipitated NbPsbO-Myc was comparable to the EV control (Figure 6A). Similar competition assays were conducted to test the relationship of NSvc4, NbPsbP, and NbPsbQ. The results showed that NSvc4, but not NSvc4_Δ181–210_, significantly reduced the amount of NbPsbP-Myc co-immunoprecipitated with NbPsbQ-FLAG compared with the EV control (Figure 6B). These results suggest that NSvc4 interferes with the interaction of NbPsbQ with both NbPsbO and NbPsbP by competitive binding to NbPsbQ. Next, we conducted BiFC assays to further validate these findings. Confocal microscopy showed that the co-expression of NSvc4 with NbPsbQ-cYFP and NbPsbO-nYFP (or NbPsbP-nYFP) resulted in markedly weaker YFP fluorescence compared with the EV control (Figure 6C,D). However, co-expression of the interaction-deficient mutant NSvc4_Δ181–210_ did not reduce the YFP signal, which remained comparable to the EV control (Figure 6C,D). Fluorescence intensity quantification confirmed that the presence of NSvc4 but not NSvc4_Δ181–210_ interferes with the interaction of NbPsbQ with NbPsbO and NbPsbP (Figure 6E,F).

## 3. Discussion

This study elucidates a mechanism by which the RSV movement protein NSvc4 subverts chloroplast-mediated immunity. We demonstrate that NSvc4 targets chloroplasts and physically interacts with the extrinsic subunit NbPsbQ to promote viral infection. This interaction inhibits defense-related cROS accumulation, and attenuates PAMP-triggered immunity (PTI)-responsive gene expression, collectively creating a cellular environment conducive to RSV infection. Our findings expand the functional repertoire of viral movement proteins and underscore the OEC as a central battleground in plant–virus interactions.

Viral movement proteins of the 30K superfamily are traditionally characterized by their ability to modify plasmodesmata and facilitate cell-to-cell transport of viral genomes [47,48,49]. The localization of RSV NSvc4 to chloroplasts adds a novel dimension to the function of this protein family. We previously confirmed that NSvc4 independently targets and enters chloroplasts via its N-terminal 20 amino acids [40]. Once inside the chloroplast, NSvc4 executes a virulence function distinct from its canonical role in movement. This is reminiscent of some potyviral P1 or geminiviral C4 proteins, which also possess multifunctional capabilities, including chloroplast targeting for immune suppression [11,12]. Our work positions NSvc4 as a key viral effector that directly bridges viral intercellular movement and intracellular immunosuppression.

Beyond photosynthetic functions, the OEC subunits are increasingly implicated in chloroplast-mediated defense [32,50]. Notably, the production of cROS, a key immune signal, is intimately linked to the photosynthetic electron transport chain where PSII and the OEC are located [51,52]. Furthermore, the extrinsic subunits of the OEC have emerged as common targets for diverse pathogens. Previous studies have shown that the downregulation of PsbO or PsbP enhances plant susceptibility to viruses such as tobacco mosaic virus and AMV [33,53]. Here, we provide evidence that PsbQ, the third member of the OEC peripheral complex, is equally vital for antiviral defense. Knockout or silencing of NbPsbQ in N. benthamiana compromises flg22-induced PR gene expression, diminishes the cROS burst in response to RSV infection, and significantly enhanced viral accumulation. These results establish NbPsbQ as a positive regulator of both basal photosynthesis and chloroplast-initiated immunity. This aligns with findings in plant-bacterial interactions, where the bacterial effector HopN1 targets and degrades PsbQ to suppress immunity [32], underscoring the conservation of PsbQ as a critical defense hub.

The molecular mechanism by which NSvc4 disrupts OEC function is particularly noteworthy. We found that NSvc4 does not block the import of NbPsbQ into chloroplasts. Instead, it acts as a molecular competitor. This is supported by the finding that binding of NSvc4 to NbPsbQ interferes with the interaction between NbPsbQ and its native partners, NbPsbO and NbPsbP, as demonstrated by both Co-IP and BiFC assays. This competition likely destabilizes the higher-order structure of the OEC, impairing its function in cROS production. This competitive inhibition mechanism contrasts with the strategy employed by the SP of RSV, which impedes the chloroplast import of PsbP [41]. Thus, RSV appears to deploy a two-pronged attack on the OEC: SP hinders the recruitment of PsbP, while NSvc4 disrupts the functional integration of PsbQ, which ensures efficient suppression of OEC-mediated defense.

**Figure 7:**
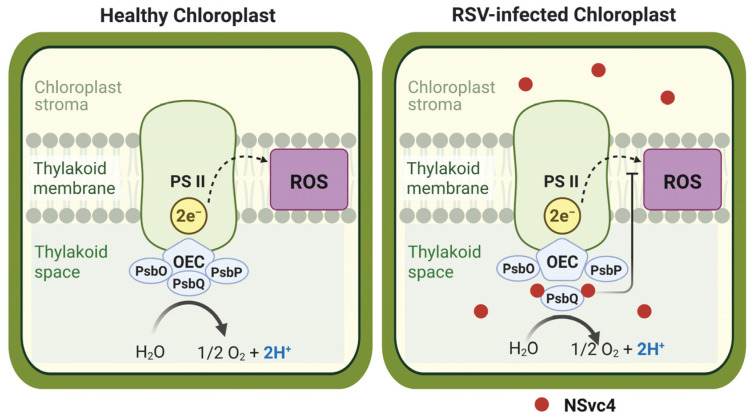
A model illustrating the molecular mechanism of the interaction between NSvc4 and NbPsbQ. The interaction between chloroplast-targeted NSvc4 and NbPsbQ inhibits the interaction between NbPsbQ and NbPsbO or NbPsbP, which consequently suppresses cROS-dependent antiviral defense. PSII: photosystem II, OEC: oxygen-evolving complex, ROS: reactive oxygen species. This figure was created in BioRender. Li, Z. (2026) (https://BioRender.com/b23sh2x, accessed on 22 April 2026).

In conclusion, we propose a refined model for RSV pathogenesis in which the movement protein NSvc4 functions as a chloroplast-targeted virulence factor. By competing with NbPsbO and NbPsbP for binding to NbPsbQ, NSvc4 destabilizes the OEC, dampens cROS signaling, and impairs chloroplast-mediated antiviral immunity (Figure 7). This study deepens our understanding of the complex interplay between viruses and chloroplasts, revealing the OEC as a critical host complex co-opted and disrupted by viral factors.

## 4. Materials and Methods

### 4.1. Plant Materials and Virus Inoculation

*N. benthamiana* plants were cultivated in a growth chamber maintained at 25 °C with 60% relative humidity, and the photoperiod was set as 16 h light and 8 h dark. Mechanical inoculation with RSV was performed as previously described [36].

### 4.2. Plasmid Construction

Total RNA was isolated with TRIzol reagent (Invitrogen, Carlsbad, CA, USA) following a previously described protocol [40]. First-strand cDNA was obtained by reverse transcription of total RNA isolated from wild-type and RSV-infected *N. benthamiana* leaves using the ReverTra Ace qPCR RT Master Mix with gDNA Remover Kit (TOYOBO, Osaka, Japan). Target genes from *N. benthamiana* and RSV were amplified via PCR using specific primer pairs and subsequently inserted into appropriate vectors. To generate expression constructs for plant transformation, the pGD-2×35S-FLAG vector was digested with *Sal*I, and the *NbPsbQ* CDS (GenBank accession number JF897611.1) and NSvc4 mutants were fused into the linearized pGD-2×35S-FLAG vector using the ClonExpress II One Step Kit (Vazyme, Nanjing, China). Similarly, the *NbPsbQ* CDS, NSvc4_Δ1–30_, and NSvc4_Δ181–210_ were fused into *BamH*I-digested pGD-2×35S-eGFP vector. The *NbPsbO* (GenBank accession no. JF897604.1) and *NbPsbP* (GenBank accession no. JF897609.1) CDS were fused into *BamH*I- and *Sac*I-digested pGD-2×35S-Myc vector. NSvc4_Δ181–210_ were fused into *BamH*I- and *Sac*I-digested pCV vector. For BiFC assays, NSvc4_Δ181–210_, *NbPsbO*, *NbPsbP*, and GUS were ligated into vectors harboring the N-terminal half of YFP (nYFP) using T4 DNA ligase (ThermoFisher, Waltham, MA, USA), while *NbPsbQ* was inserted into vectors carrying the C-terminal half of YFP (cYFP) following the same procedure. Primer sequences are listed in Table S1.

### 4.3. Transient Expression Assay in N. benthamiana Plants

Transient expression experiments were conducted according to established methods [40]. In brief, the expression constructs were introduced into *Agrobacterium tumefaciens* strains EHA105 via electroporation. The resulting agrobacterial cells were suspended in infiltration buffer (10 mM MgCl_2_, 10 mM MES, pH 5.6, and 150 μM acetosyringone) to a final OD_600_ of 0.6–0.8. Prior to infiltration, the bacterial suspensions were incubated at room temperature for 2 h. Five-week-old *N. benthamiana* plants were used for agroinfiltration. For co-expression experiments, suspensions carrying distinct expressing vectors were combined at a 1:1 ratio before infiltration.

### 4.4. qRT-PCR

Gene-specific primer pairs were designed with Oligo 7 software (https://www.oligo.net/, accessed on 2 May 2023). qRT-PCR was carried out on a LightCycler 480 (Roche, Basel, Switzerland) using ChamQ Universal SYBR qPCR Master Mix (Vazyme, Nanjing, China) as described previously [40].

### 4.5. Western Blot Analysis

Total plant protein was extracted with protein extraction buffer following a previously published protocol [36]. In brief, leaf samples (0.1 g) were ground with liquid nitrogen, and suspended in 200 μL extraction buffer (9 M urea, 4.5% sodium dodecyl sulfate, 1.5% β-mercaptoethanol, 50 mM Tris-HCl pH 6.8). Then, the mixtures were centrifuged at 12,000× *g* for 10 min, and the resultant supernatants were heated at 80 °C for 5 min. Equal protein amounts were resolved by 12.5% SDS-PAGE and transferred onto nitrocellulose membranes. Blots were then incubated with specific primary antibodies recognizing GFP (ABclonal, Wuhan, China), the FLAG epitope tag (Sigma, St. Louis, MO, USA), the Myc tag (GenScript, Nanjing, China), and actin (ABclonal, Wuhan, China). Primary antibodies directed against RSV NSvc4 and RSV CP were produced in our lab.

### 4.6. Protoplast Isolation and Visualization of Cellular ROS

*N. benthamiana* leaves were sliced into small segments and incubated in enzyme solution following a prior description [36]. After a 2 h incubation, protoplasts were observed under a confocal microscope. H_2_DCF-DA (2′7′-dichlorodihydrofluorescein diacetate) served as a probe to monitor photosynthesis-derived ROS in *N. benthamiana* protoplasts, as previously reported [54]. In brief, 10 μM H_2_DCF-DA (Sigma-Aldrich, St. Louis, MO, USA) was infiltrated into *N. benthamiana* leaves or added to protoplasts, and one hour later, the DCF fluorescence was monitored using a confocal microscope with excitation at 488 nm and emission collected at 512–527 nm.

### 4.7. Subcellular Localization Assay and Quantification of Fluorescence Intensity

The eGFP fluorophore was excited at 488 nm laser lines, and emitted at 490–540 nm. The YFP fluorophore was excited at 514 nm laser lines, with emission detected at 520–540 nm. Chlorophyll was excited using the 488 nm laser line, and its emission was captured at 640–720 nm. Images were acquired with an FV3000 laser scanning confocal microscope (Olympus, Tokyo, Japan), and the intensity of fluorescence was analyzed with cellSens software v4.2 (Olympus) as previously described [40].

### 4.8. Co-IP Assay

Total protein from different combinations were extracted using IP buffer (40 mM Tris-HCl, pH 7.5, 150 mM NaCl, 5 mM MgCl_2_, 2 mM EDTA, 5 mM DTT, 1% Triton X-100, 5% glycerol) supplemented with MedChemExpress Protease Inhibitor Cocktail (EDTA-free, mini-tablet, 1 tablet per 10 mL). The protein mixture was incubated at 4 °C for 30 min, and centrifuged twice at 12,000× *g* for 15 min at 4 °C. The clarified lysate was immunoprecipitated using anti-FLAG M2 magnetic beads (Sigma-Aldrich, St. Louis, MO, USA). Subsequent steps were carried out as previously described [40].

## Data Availability

The original contributions presented in this study are included in the article/Supplementary Materials. Further inquiries can be directed to the corresponding author.

## Supplementary Materials

The following supporting information can be downloaded at: https://www.mdpi.com/article/10.3390/ijms27093859/s1.
