# Supplementary material for: The NSvc4 Protein of Rice Stripe Virus Suppresses Chloroplast-Mediated Defense by Interacting with NbPsbQ
Source: Int J Mol Sci. 2026 Apr 27;27(9):3859. doi: 10.3390/ijms27093859 (PMC13164279; doi:10.3390/ijms27093859)
Supplement: Supplementary file 1 [file ijms-27-03859-s001.zip › ijms-4243151-supplementary.pdf]

Supplementary Information

Table S1 Primers used in this study

Primers	Sequences (5’-3’)
**Primers used for qRT-PCR**	
**Primers**	**Sequences (5’-3’)**
NbActin-F	AAGCTGCAGGTATCCATGAGACTA
NbActin-R	CAATCCAGACACTGTACTTTCTCTC
NbPR1-F	GGATGCCCATAACACAGCTC
NbPR1-R	GCCTTAGCAGCCGTCATGA
NbPR2-F	GGGCTGTTAATTTGCAGTATCC
NbPR2-R	GGTTTATAACATCTTGGTCTGATGG
RSV CP-F	AGTGCTGATCGTATTGACAGA
RSV CP-R	GATGAAGTACACAACTGGTC
NbPsbQ-FLAG-F	AGAACACGGGGGACGAGCTCATGGCTCATGCTATGGCTTC
NbPsbQ-FLAG-R	GTGGTCCTTATAGTCGTCGACACCAAGTTTGGCCAAAACATC
NSvc4$_{\Delta 1-30}$-FLAG-F	GAGCTCGGTACCCGGATGAATAGGAAATCTCTAGCTCTCTCC
NSvc4$_{\Delta 1-30}$-FLAG-R	TTATAGTCGTCGACTCTAGACATGATGACAGAAACTTCAG
NSvc4$_{\Delta 31-60}$-FLAG-F	AAGAGAGTTGATAATAAGTTCAGCTTCTCAGATGTC
NSvc4$_{\Delta 31-60}$-FLAG-R	CTTATTATCAACTCTCTTTTGGGACTC
ofNSvc4$_{\Delta 61-90}$-FLAG-F	CAATGCTTGGATTGGAACCTAACATACTTGTTGATCCATATTGG
NSvc4$_{\Delta 61-90}$-FLAG-R	AGGTTCCAATCCAAGCATTG
NSvc4$_{\Delta 91-120}$-FLAG-F	CCCATAGGAAAGCAACTTTCATTAAGGGTAGAGCTAGTGGAAT
NSvc4$_{\Delta 91-120}$-FLAG-R	GAAAGTTGCTTTCCTATGGGCC
**Primers used for construction of different vectors**	
**Primers**	**Sequence (5’-3’)**
NSvc4$_{\Delta 121-150}$-FLAG-F	CTATGGTCTGGATTGGATACCCAATTAGCAAGAATTTTGC
NSvc4$_{\Delta 121-150}$-FLAG-R	TCCAATCCAGACCATAGCA
NSvc4$_{\Delta 151-180}$-FLAG-F	GTTGAGGTAGAAGTTCGCGTTGATGACAGCTCAGTGC
NSvc4$_{\Delta 151-180}$-FLAG-R	GCGAACTTCTACCTCAACTTG
NSvc4$_{\Delta 181-210}$-FLAG-F	CTTCAAGTGTCCGTATCTACTAATGACACTGTGATGTTTG

NSvc4$_{\Delta 181\text{-}210}$-FLAG-R	AGATACGGACACTTGAAGATTATG
NSvc4$_{\Delta 211\text{-}240}$-FLAG-F	CTGCCAGTGTCCATGAAGGTTCAGAAAGCTGTTGGAGG
NSvc4$_{\Delta 211\text{-}240}$-FLAG-R	CTTCATGGACACTGGCAGA
NSvc4$_{\Delta 241\text{-}270}$-FLAG-F	GTAATTTTACGACTAATGTTAAGATACCCATCACAAAGAAATC
NSvc4$_{\Delta 241\text{-}270}$-FLAG-R	AACATTAGTCGTAAAATTACTGAAGC
NSvc4$_{\Delta 271\text{-}286}$-FLAG-F	GTGGTGAAGCAGCCAAAGTCTAGAGTCGACGACTATAAGG
NSvc4$_{\Delta 271\text{-}286}$-FLAG-R	CTTTGGCTGCTTCACCACAC
NbPsbO-Myc-F	TTTACGAACGATAGGGATCCATGGCTACCTCTCTACAAGC
NbPsbO-Myc-R	ATGAGTTTCTGCTCGAGCTCTTCAAGTTGGGCATACCAGAT
NbPsbP-Myc-F	TACGAACGATAGGGATCCATGGCTTCAACACAATGTTTCT
NbPsbP-Myc-R	ATGAGTTTCTGCTCGAGCTCAGCAACACTGAAAGAAGTGG
pCV-NSvc4$_{\Delta 181\text{-}210}$-F	ACTCTAGACCCCTGGGATCCATGGCTTTGTCTCGACTTTTG
pCV-NSvc4$_{\Delta 181\text{-}210}$-R	CGGGGAATTCGAGCTCTTACATGATGACAGAAACTTCAGATTTTG
NSvc4$_{\Delta 181\text{-}210}$-nYFP-F	TACGAACGATAGTTAATTAAATGGCTTTGTCTCGACTTTTG
NSvc4$_{\Delta 181\text{-}210}$-nYFP-R	CTCCACTAGTGGCGCGCCCCATGATGACAGAAACTTCAGATTTTG
NbPsbO-nYFP-F	TACGAACGATAGTTAATTAAATGGCTACCTCTCTACAAGC
NbPsbO-nYFP-R	CTCCACTAGTGGCGCGCCCTTCAAGTTGGGCATACCAGAT
NbPsbP-nYFP-F	ACGAACGATAGTTAATTAAATGGCTTCAACACAATGTTTC
NbPsbP-nYFP-R	CCTCCACTAGTGGCGCGCCCAGCAACACTGAAAGAAGTGG
NbPsbQ-cYFP-F	TACGAACGATAGTTAATTAAATGGCTCATGCTATGGCTTC
NbPsbQ-cYFP-R	CCTCCACTAGTGGCGCGCCCACCAAGTTTGGCCAAAACAT

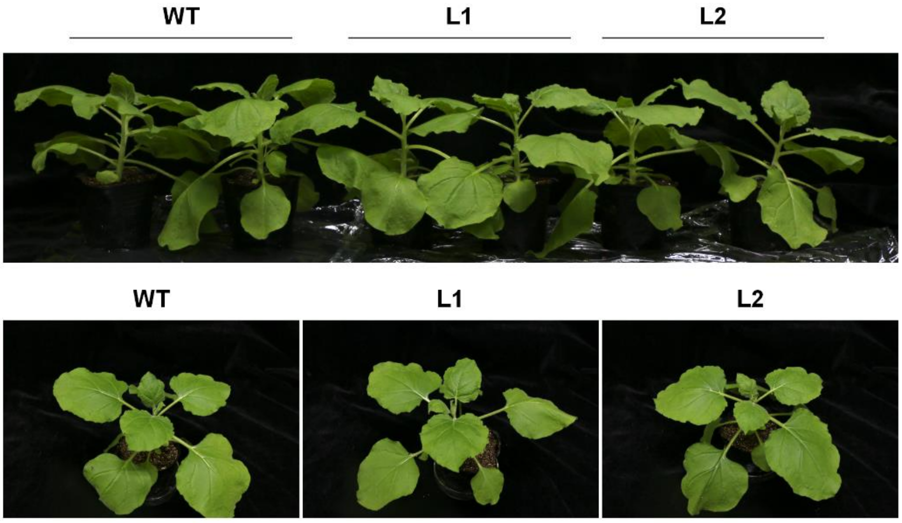

Figure S1. The phenotype of *Nbpsbq* knock-out *N. benthamiana* plants.

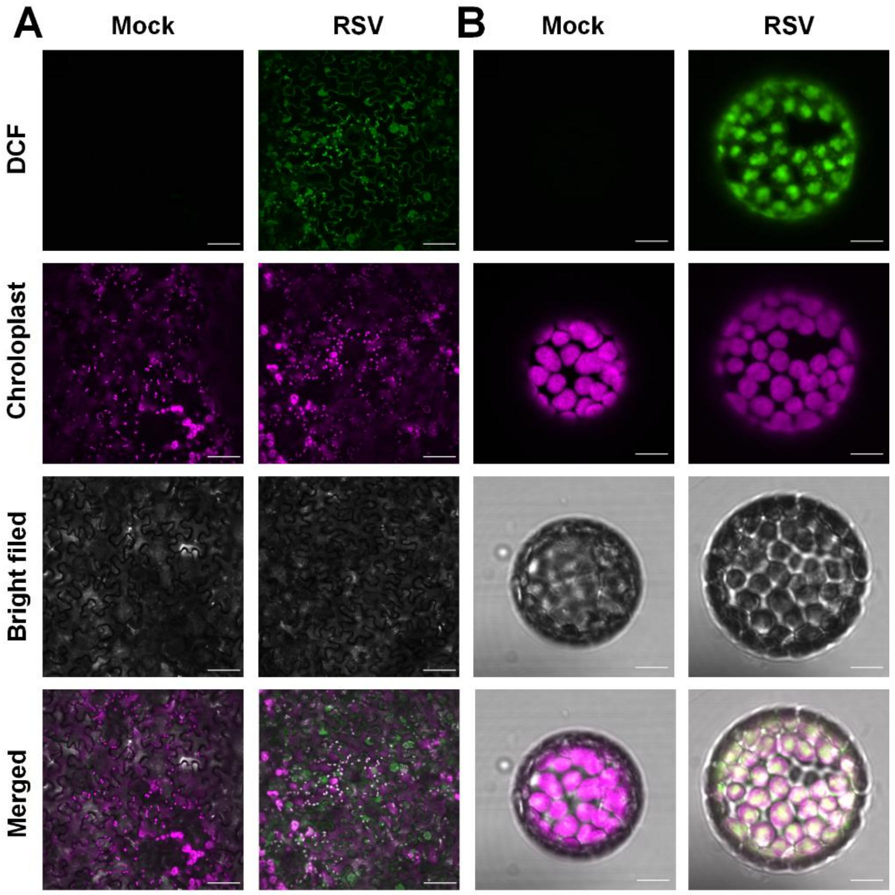

Figure S2. RSV infection leads to the accumulation of ROS in chloroplasts

(A) Confocal microscopy of the subcellular localization of DCF in the leaves of healthy and RSV-infected *N. benthamiana* plants. Bars, 50 µm. (B) Subcellular localization of DCF in the protoplasts of healthy and RSV-infected *N. benthamiana* plants. Bars, 5 µm.

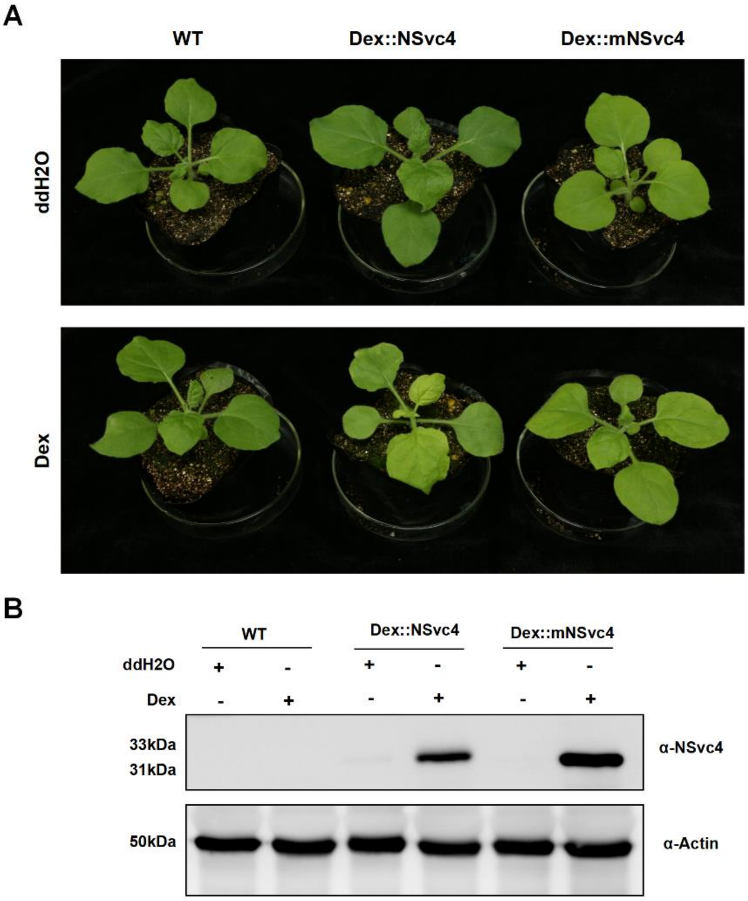

Figure S3. Phenotype analysis of Dex::NSvc4 and Dex::mNSvc4 transgenic plants

(A) Phenotype observation of Dex::NSvc4 and Dex::mNSvc4 transgenic plants induced by Dex. (B) Western blot analysis of the expression level of NSvc4 and mNSvc4 induced by Dex.

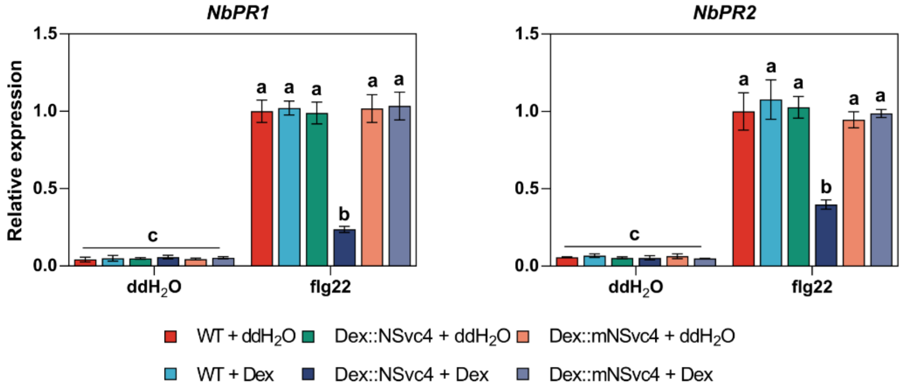

Figure S4. Dex-induced expression of NSvc4 inhibits the expression of *PR* genes

The relative expression levels of *NbPR1* and *NbPR2* in WT, Dex::NSvc4 and Dex::mNSvc4 plants treated with ddH2O or Dex after infiltration with flg22 were analyzed by qRT-PCR with the *NbActin* as a reference gene for normalization. The relative expression levels were normalized by setting the value of WT + H_2O treated with flg22 as 1. Data are presented as mean ± SD ($n = 3$). One-way ANOVA followed by Tukey's HSD post hoc test was performed for multiple comparisons. Different letters indicate statistically significant differences between groups ($P < 0.05$). Three independent replicates were performed and comparable results were obtained.

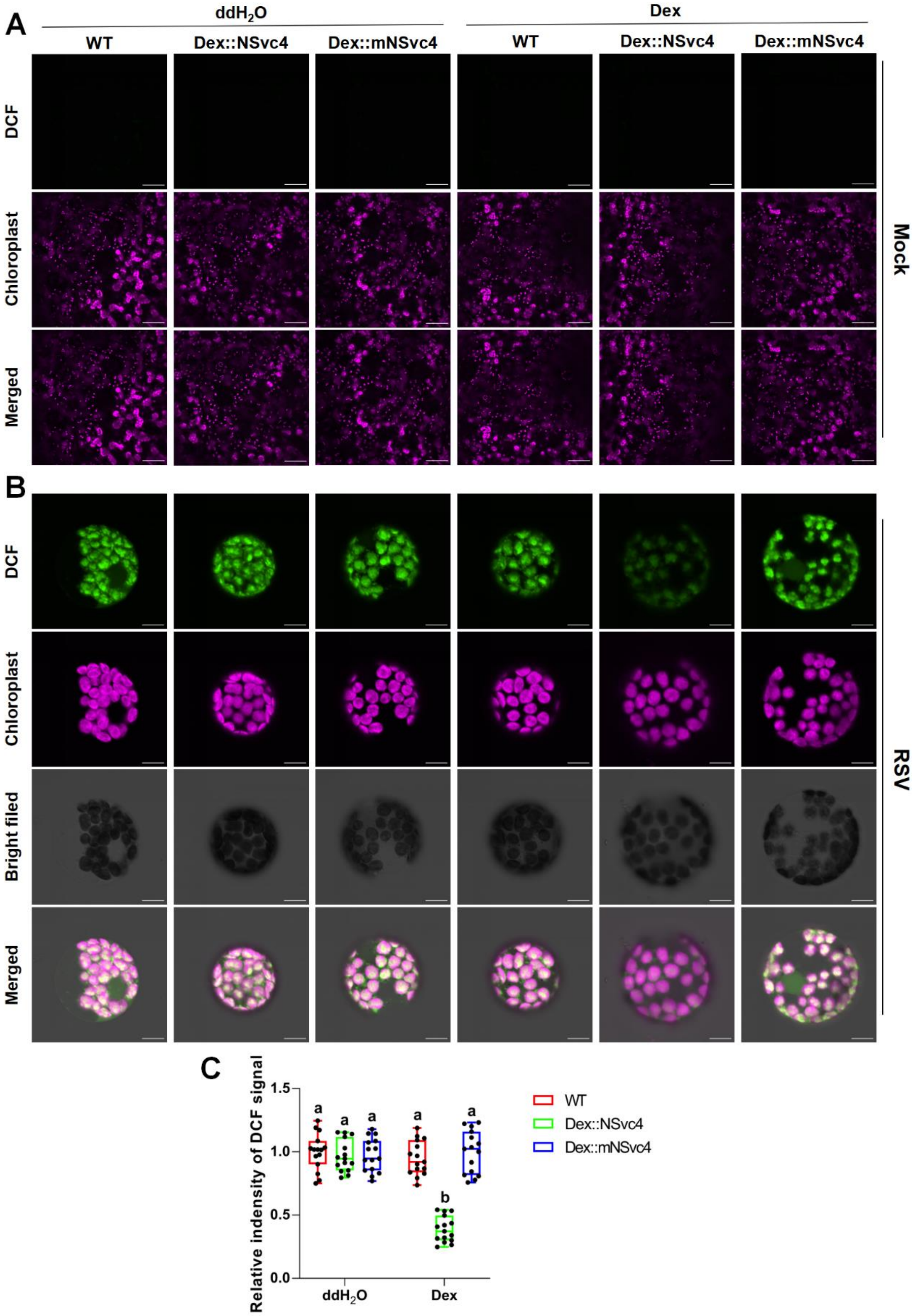

Figure S5. Expression of NSvc4 inhibits the accumulation of cROS during RSV infection

(A) Detection of cROS in WT, Dex::NSvc4 and Dex::mNSvc4 plants. The upper leaves of these plants treated with ddH_2O or Dex were injected with 10 mM H_2DCF-DA. 1

hour later, the subcellular localization of DCF was observed under a confocal microscope. Bars, 50 µm. (B) Detection of cROS in RSV-infected WT, Dex::NSvc4 and Dex::mNSvc4 plants. WT, Dex::NSvc4 and Dex::mNSvc4 plants treated with ddH_2O or Dex were mechanically inoculated with RSV. At 7 dpi, 10 mM H_2DCF-DA was added to protoplasts isolated from RSV-infected *N. benthamiana* leaves. 1 hour later, the subcellular localization of DCF was observed under a confocal microscope. Bars, 5 µm. (C) Quantification of DCF fluorescence intensity in chloroplasts of (B). The relative intensity of DCF signal were normalized by setting the value of WT treated with ddH_2O as 1. One-way ANOVA followed by Tukey's HSD post hoc test was performed for multiple comparisons. Different letters indicate statistically significant differences between groups (n = 15, $P < 0.05$). Each experiment was performed three times independently with comparable results.

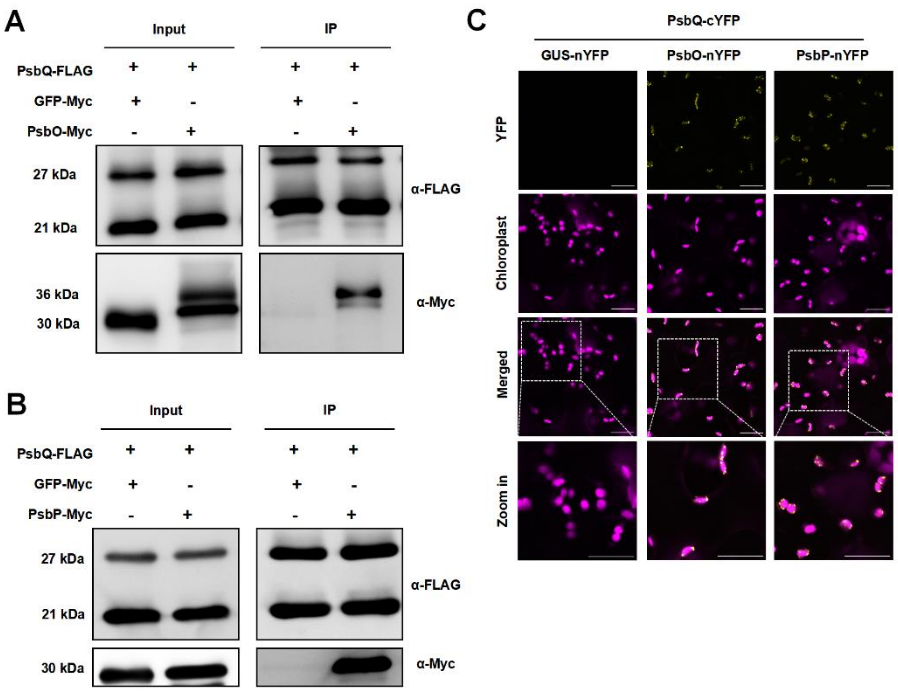

Figure S6. Analysis of interaction of NbPsbQ with NbPsbO and NbPsbP

(A & B) Co-IP analysis of the interactions between NbPsbQ and NbPsbO, NbPsbP. (C) BiFC analysis of the interaction. Bars, 20 μm.
